# Supplementary material for: Egr2 drives the differentiation of Ly6Chi monocytes into fibrosis-promoting macrophages in metabolic dysfunction-associated steatohepatitis in mice
Source: Commun Biol. 2024 Jun 3;7:681. doi: 10.1038/s42003-024-06357-5 (PMC11148031; doi:10.1038/s42003-024-06357-5)
Supplement: Supplementary file 2 — Supplementary Information [file 42003_2024_6357_MOESM2_ESM.pdf]

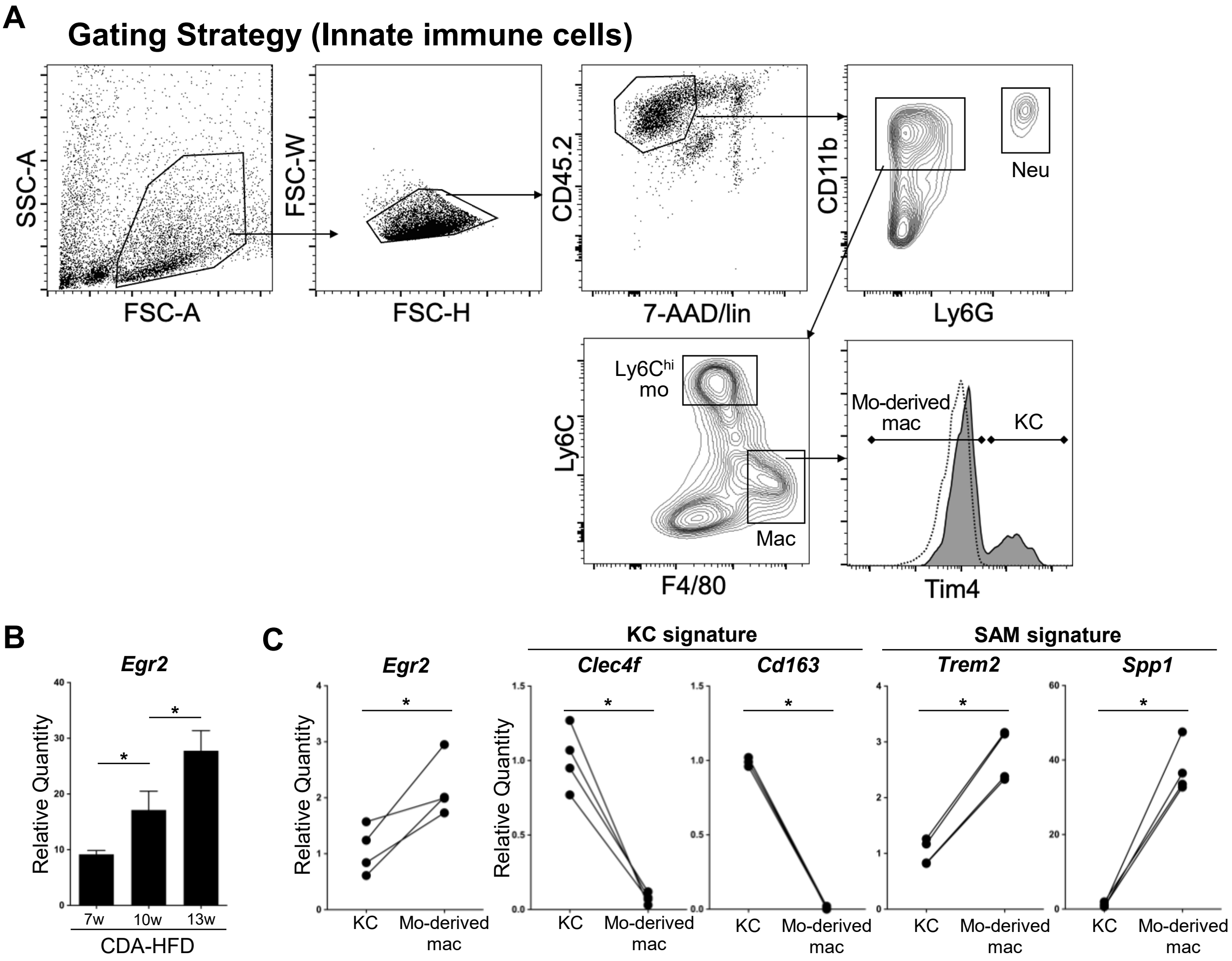

Supplementary Figure 1. Characterization of resident KCs and mo-derived mac in MASH liver.

(**A**) Gating strategy of liver innate immune cells from mice fed CDA-HFD for 4 weeks. Lin includes B220, CD90.2, NK1.1, and SiglecF. Neu, neutrophils; mo, monocytes; mac, macrophages; KC, Kupffer cells. Dashed line represents Tim4 unstained control. (**B**) *Egr2* mRNA expression by Ly6C^{lo}F4/80^{+} macrophages was positively correlated with the progression of MASH. Average quantities relative to ND-fed mice are shown with SD. n = 2 mice/ND, n = 3 mice/7 weeks, 10 weeks, and 13 weeks. *$p < 0.05$, one-way ANOVA. (**C**) qPCR analysis of Tim4^{+} resident KCs and Tim4^{-} mo-derived mac from mice fed CDA-HFD for 4 weeks. *Egr2* mRNA is more strongly expressed in Tim4^{-} mo-derived mac than in Tim4^{+} KCs. n = 4 mice/cell type. *$p < 0.05$, paired t-test.

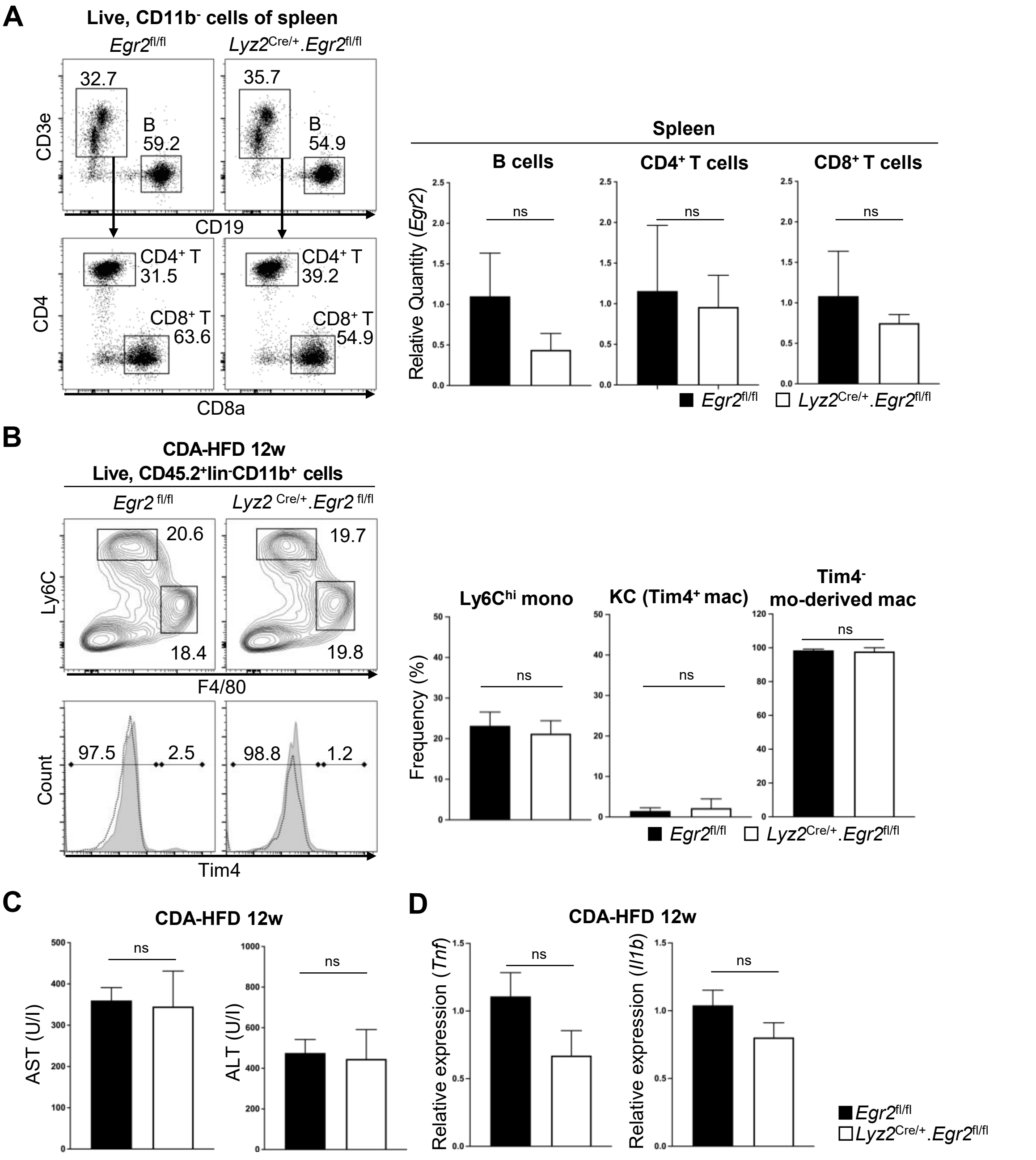

Supplementary Figure 2. Evaluation of immune cells in mice lacking *Egr2*.

(**A**) Gating strategy of spleen lymphocytes from *Egr2*$^{fl/fl}$ mice and *Lyz2*$^{Cre/+}$.*Egr2*$^{fl/fl}$ mice (left). *Egr2* gene expression by lymphocytes is comparable between 2 genotypes of mice (right). Filled bar, *Egr2*$^{fl/fl}$; empty bar, *Lyz2*$^{Cre/+}$.*Egr2*$^{fl/fl}$. n = 3 mice/genotype. Means +/- SD are shown. (**B**) Percentages of liver monocytes and macrophages in CD45.2^{+}lin^{-} CD11b^{+} cells from *Egr2*$^{fl/fl}$ and *Lyz2*$^{Cre/+}$.*Egr2*$^{fl/fl}$ mice fed CDA-HFD for 12 weeks (left). Lin includes CD90.2, B220, NK1.1, SiglecF, and Ly6G. Representative FACS plots of 3-4 mice are shown. Histogram plot showing the proportions of Tim4^{+} resident KCs and Tim4^{-} mo-derived mac in Ly6C^{lo}F4/80^{+} cells (bottom left). Dashed line indicates Tim4 unstained control. Averages of 5-6 mice/genotype are shown with SD (right). (**C**) Serum AST and ALT concentrations in *Egr2*$^{fl/fl}$ mice and *Lyz2*$^{Cre/+}$.*Egr2*$^{fl/fl}$ mice fed CDA-HFD for 12 weeks. n = 3 mice/genotype. Means +/- SD are shown. (**D**) Whole-liver qPCR analysis of *Tnf* in *Egr2*$^{fl/fl}$ and *Lyz2*$^{Cre/+}$.*Egr2*$^{fl/fl}$ mice fed CDA-HFD for 12 weeks. n = 7-9 mice/genotype. Mean quantities relative to *Egr2*$^{fl/fl}$ mice are shown with SEM; *$p < 0.05$; ns, not significant; t-test.

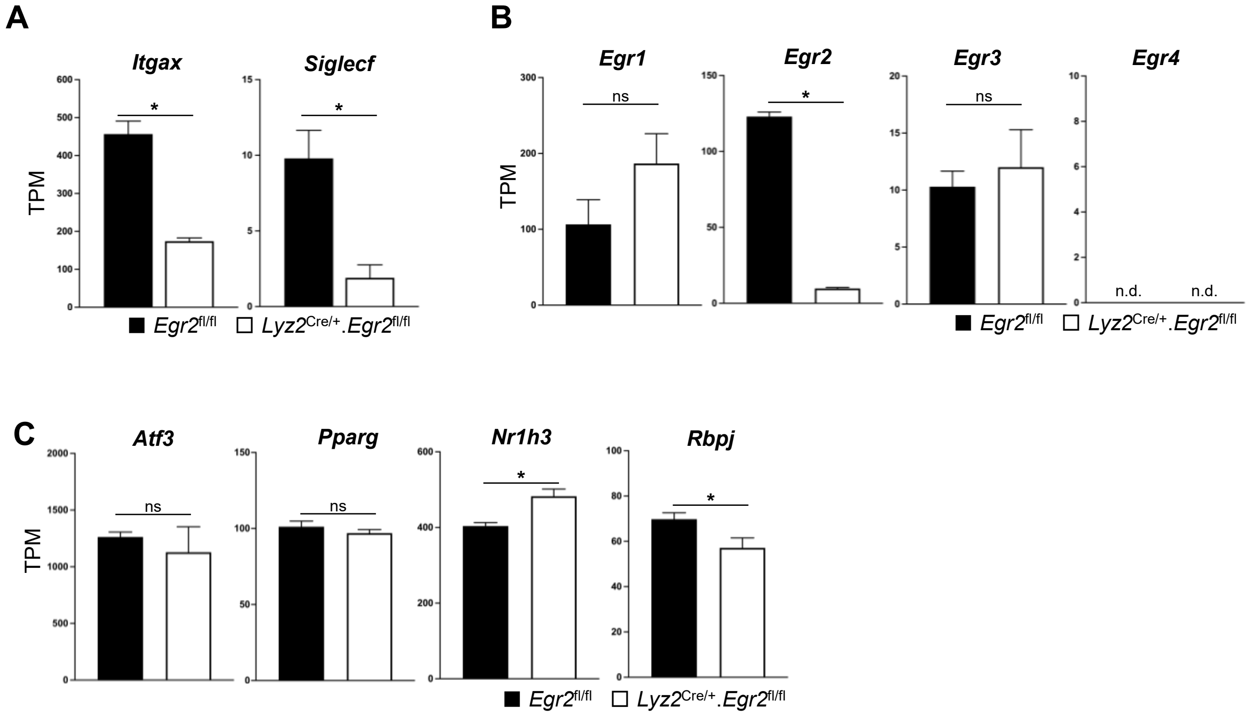

Supplementary Figure 3. Transcriptomic changes in macrophages of *Egr2*-deficient mice in MASH liver.

(**A**) Expression levels of known downstream targets of Egr2 (*Itgax* and *Siglecf*). TPM, transcripts per kilobase million. Filled bar, *Egr2*$^{fl/fl}$; empty bar, *Lyz2*$^{Cre/+}$.*Egr2*$^{fl/fl}$. n = 3 mice/genotype. Means +/- SD are shown. *$p < 0.05$, t-test. (**B**) Bar graphs showing gene expression levels of EGR family (*Egr1*, *Egr2*, *Egr3*, and *Egr4*) in liver macrophages. n = 3 mice/genotype. Means +/- SD are shown. (**C**) Bar graphs showing gene expression levels of some transcriptional factors known to control liver macrophage differentiation. n = 3 mice/genotype. Means +/- SD are shown. TPM, transcripts per kilobase million. Filled bar, *Egr2*$^{fl/fl}$; empty bar, *Lyz2*$^{Cre/+}$.*Egr2*$^{fl/fl}$. n.d., not detectable. *$p < 0.05$, t-test.

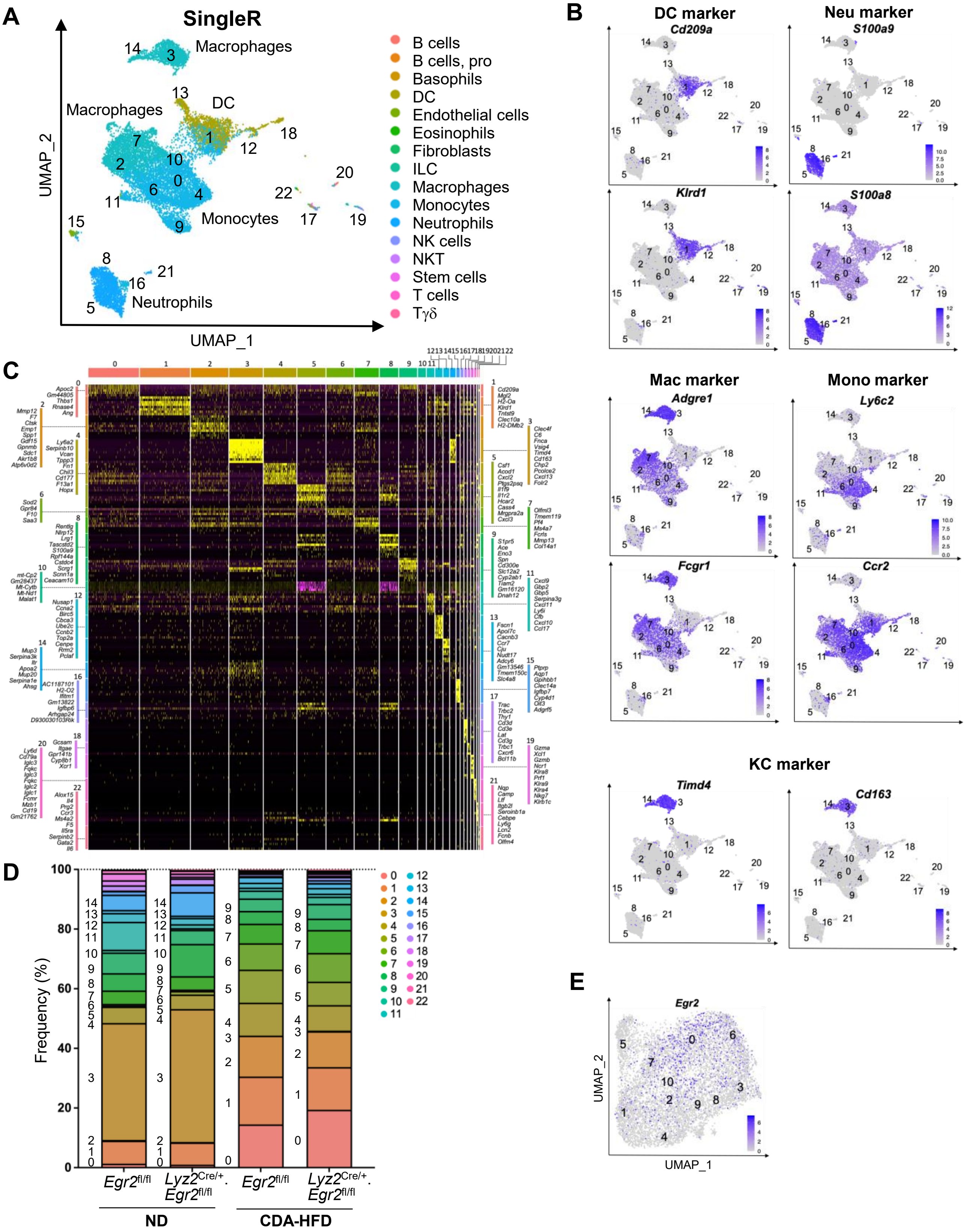

Supplementary Figure 4. Single cell characterization of the liver immune cells in MASH. (**A**) Unbiased annotation of clusters defined by UMAP plots by using SingleR. (**B**) Heat maps of marker genes that were used to manually annotate UMAP clusters. (**C**) Heatmap showing top 10 discriminative genes per cluster defined in **Fig 4A**. (**D**) Proportion of clusters defined by UMAP plots in **Fig 4A**. (**E**) Heat map showing *Egr2* expression defined in **Fig 4B**.

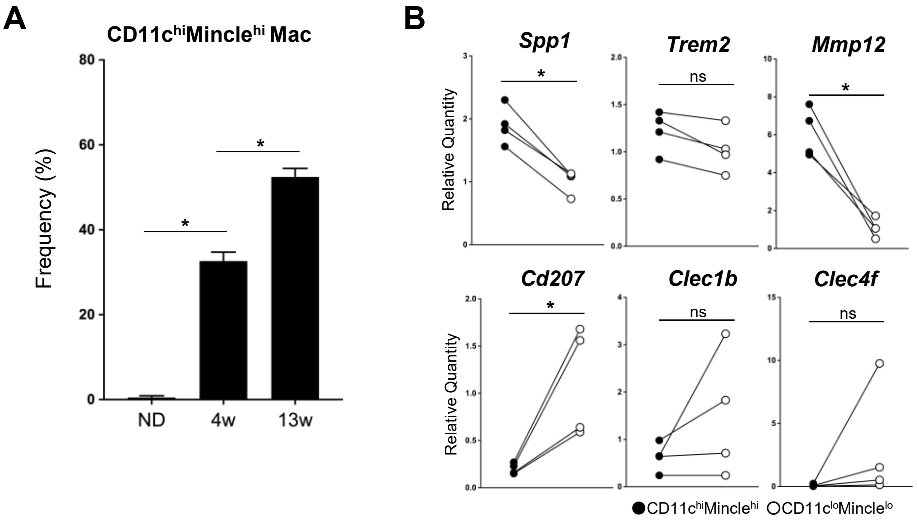

Supplementary Figure 5. Mo-derived mac can be divided into hLAMs and moKCs on the basis of differential CD11c and Mincle expression.

(**A**) Proportion of CD11c^{hi}Mincle^{hi} macrophages in CD45.2^{+}lin^{-}CD11b^{+}Ly6C^{lo}F4/80^{+} cells progressively increased during the development of MASH. Lin includes CD90.2, B220, NK1.1, SiglecF, and Ly6G. n = 3 mice/timepoint. Means +/- SD are shown. *$p < 0.05$, one-way ANOVA. (**B**) Gene expression levels of indicated hLAM signatures (top) and moKC signatures (bottom) were measured by qPCR. n = 4/cell type. Average expression levels relative to CD11c^{lo}Mincle^{lo} macrophages are shown. Each symbol represents an individual animal. *$p < 0.05$; ns, not significant; paired t-test.
