# Supplementary material for: Egr2 drives the differentiation of Ly6Chi monocytes into fibrosis-promoting macrophages in metabolic dysfunction-associated steatohepatitis in mice
Source: Commun Biol. 2024 Jun 3;7:681. doi: 10.1038/s42003-024-06357-5 (PMC11148031; doi:10.1038/s42003-024-06357-5)
Supplement: Supplementary file 3 — Description of Additional Supplementary Files [file 42003_2024_6357_MOESM3_ESM.docx]

Description of Additional Supplementary Files

**File name:** Supplementary Data 1

**Description:** Genes differentially expressed between two macrophages (cluster 3 and cluster 6) from MASH liver.

**File name:** Supplementary Data 2

**Description:** Expression of genes associated with cytokine activity (GO:0005125) in naïve liver and MASH liver.

**File name:** Supplementary Data 3

**Description:** Gene sets used for Gene Set Enrichment Analysis (GSEA).

**File name:** Supplementary Data 4

**Description:** Gene sets used for AddModuleScore analysis.

**File name:** Supplementary Data 5

**Description:** The source data behind the graphs in this paper.
